# Do Financial Literacy and Financial Education Influence Smoking Behavior in the United States?

**DOI:** 10.3390/ijerph18052579

**Published:** 2021-03-04

**Authors:** Mostafa Saidur Rahim Khan, Pongpat Putthinun, Somtip Watanapongvanich, Pattaphol Yuktadatta, Md. Azad Uddin, Yoshihiko Kadoya

**Affiliations:** 1 School of Economics, Hiroshima University, 1-2-1 Kagamiyama, Higashihiroshima, Hiroshima 739-8525, Japan; putthinun@gmail.com (P.P.); somtip.w@gmail.com (S.W.); d206295@hiroshima-u.ac.jp (P.Y.); ykadoya@hiroshima-u.ac.jp (Y.K.); 2 Graduate School for International Development and Cooperation, Hiroshima University, 1-5-1 Kagamiyama, Higashihiroshima, Hiroshima 739-8529, Japan; d193319@hiroshima-u.ac.jp

**Keywords:** smoking, financial literacy, financial education, rationality, United States

## Abstract

Smoking is still a serious economic, health, and social problem despite various efforts to curb its prevalence. We examined the influence of financial literacy and financial education on the smoking behavior in the United States in terms of the use of rational decision-making abilities to reduce irrational behavior. We hypothesized that financial literacy and financial education, as proxies for rational decision making, would reduce the likelihood of smoking. We used data from the Preference Parameters Study (PPS) of Osaka University conducted in the United States in 2010 and applied probit regression models to test our hypothesis on a sample of 3831 individuals. We found that financially literate people are less likely to be smokers, though we found no clear role of financial education in reducing the likelihood of smoking. Further, respondents’ gender, age, unemployment status, and risky health behaviors such as drinking and gambling, have a significantly positive association with smoking, while marital status, university degree, family size, household income, household assets, physical exercise, and level of happiness have a significantly negative association. Our findings suggest that financial literacy, as an instrument encouraging rational decision making, could be a tool to help reduce smoking in the United States.

## 1. Introduction

A recent study found that financial literacy and financial education significantly impact the smoking behavior in Japan [1]. The study claims that financially literate and financially educated people, who have the ability to make rational decisions, are less likely to smoke because they rationally value the current pleasure and potential negative consequences of smoking. The study’s findings motivated us to investigate whether financial literacy and financial education can reduce smoking in different cultural settings, such as the United States. Culture is an essential consideration in the context of smoking behavior because prior studies provided evidence that cultural attributes such as shared values and social norms affect health-related behaviors [2,3], including tobacco use [4]. Peer pressure, which is a strong proxy for the socio-cultural environment, influences smoking initiation, continuation [5,6,7,8], cessation, and relapse [9,10,11]. A meta-analysis revealed that peer influence has stronger associations with smoking behavior in countries with collectivistic cultures than in those with individualistic cultures [6]. Omori, Yamawaki, and McKyer [12] found that the peer effect on smoking behavior in Japan’s collectivistic society is positively significant; in comparison, peer pressure in the individualistic American society has an insignificant influence on smoking behavior. Japan and the United States also differ in other socio-cultural dimensions [13,14,15]. Hence, the difference in cultural contexts raises the question of whether the empirical findings in Japan imply the same conclusion in the United States.

The adverse health effects of smoking have been extensively studied [16]. Numerous studies demonstrated that exposure to both first- and second-hand smoke can increase the risk of having a wide range of chronic physical and mental illnesses (e.g., [16,17,18]) and reduce life expectancy [19], which ultimately increases health care utilization and cost [16,20]. In addition, smoking has other behavioral and economic implications. Smoking-related illnesses are associated with absenteeism and presenteeism, leading to a decrease in work performance and productivity [21,22,23]. Because of the adverse consequences of tobacco, both public and private sectors in the United States introduced various countermeasures such as increasing the federal tobacco tax [24], imposing state taxes on tobacco [25], campaigning against smoking on mass media outlets [26], increasing the federal minimum age to purchase tobacco [27,28], banning smoking in public [29], and enhancing the access to tobacco cessation counseling and medications [26]. Consequently, the incidence of adult smoking reduced from 20.9% in 2005 to 13.7% in 2018 [30,31]. However, the latest data reveal that over 34 million people in the United States still smoke [31] and tobacco smoking is still the major reason behind preventable death and chronic disease in the United States [16,32], despite continued efforts to curtail smoking.

We observe smoking from the perspective of the imperfectly rational addiction framework and the irrational choice framework and propose a solution based on rational decision-making abilities in line with Watanapongvanich, Khan, Putthinun, Ono, and Kadoya [1]. According to Grossman [33,34], we can perceive smoking as a disinvestment in health capital and a catalyst for productivity loss and utility loss. Despite its obvious negative consequences, smoking behaviors may persist due to cognitive limitations [35,36,37,38,39,40], which influence imperfectly rational individuals to underestimate the risk of smoking [41,42]. Further, the existing literature suggests that personal emotions and social influences, especially from family members and peer groups, affect the propensity to smoke [6,7,8,9,10,11,16,43,44,45,46,47]. Preventing people from making irrational smoking decisions by enhancing the rational decision-making ability through the provision of rational decision-making instruments such as financial literacy and financial education seems to be an effective solution. Empirically, financial literacy helps people make more informed economic and financial decisions and commit to more rational behavior [48,49,50,51,52,53,54,55,56,57,58]. Moreover, financial literacy is related to improved cognitive ability [59,60,61], eventually helping people make more accurate judgments. Thus, financially literate people are less likely to engage in smoking because they can estimate the gross benefit of smoking more accurately and refrain from making decisions driven by emotional bias and social influences.

This study examines whether having financial literacy and financial education prevents one from committing irrational behaviors such as smoking. We hypothesize that financial literacy and financial education, which enable people to make rational decisions, reduce the likelihood of being a smoker. Watanapongvanich, Khan, Putthinun, Ono, and Kadoya’s [1] findings on financial literacy and financial education as rational decision-making instruments support our hypothesis. To the best of our knowledge, this is the first study which addresses the association between financial literacy, financial education, and smoking behavior among the American population. Our study provides additional substantiation to the existing literature as we report empirical evidence on the association between financial literacy and financial education as rational decision-making tools and how they affect irrational decisions related to smoking behavior in the United States. The results of this study can assist policymakers in the United States in implementing effective means of evidence-based interventions at the national, state, and local levels to prevent and minimize the negative consequences of smoking.

## 2. Materials and Methods

### 2.1. Data

#### 2.1.1. Data

We used data from the Preference Parameters Study (PPS) conducted by the Institute of Social and Economic Research at Osaka University for this study. The PPS is a panel survey that collects information on socioeconomic characteristics and preferences of individuals in Japan, the United States, China, and India. In this study, we utilized data from the 2010 wave of the survey conducted in the United States, which contained questions about smoking behavior, financial literacy, and financial education. The respondents were from the District of Columbia and 48 other states, excluding Alaska and Hawaii, and are representative of the population of the United States. Although the PPS was continued even after 2010, the later waves did not include questions on financial literacy. As a result, we used the 2010 waves, which included all the data required for this study. The sample comprised 3831 individuals, or approximately 54% of the total respondents surveyed in 2010 (7046 individuals). The PPS included several financial questions such as household income, balance of financial assets, financial literacy, financial education etc., which many respondents did not respond to. Moreover, several respondents did not respond to some demographic, socio-economic, and risky health behavior questions. As a result, we excluded several observations due to missing values on smoking behavior, financial literacy, financial education, and demographic variables from the sample (3215 observations). We believe that the excluded sample would not make the results of this study biased because the exclusion was not made on a particular issue and did not have a specific pattern.

#### 2.1.2. Variable Definitions

The dependent variable in this study was smoking behavior. The PPS contains the question “Do you smoke?” and provides seven responses, where 1 means “do not smoke at all”, 2 means “hardly smoke”, 3 means “smoke sometimes”, 4 means “about 10 cigarettes a day”, 5 means “about a pack a day”, 6 means “more than 2 packs a day”, and 7 means “I used to smoke but have quit”. We grouped these responses into a binary scale of non-smokers and current smokers by coding respondents who answered 1, 2, or 7 as 0 or non-smokers [1,62], and those who answered 3, 4, 5, or 6 as 1 or current smokers.

There are two main variables of interest in our study: financial literacy and financial education. We measured financial literacy using Lusardi and Mitchell’s [63] methodology, which is simple and widely adopted in the existing literature (e.g., [48,53,64,65,66,67,68,69,70,71]). Moreover, Nicolini and Haupt [72] found that the Lusardi–Mitchell measurement of financial literacy performs well unless financial literacy is used to explain financial behavior. The three questions which we used to measure financial literacy are as follows:

a. Suppose you had $100 in a savings account and the interest rate was 2% per year. You never withdraw money or receive interest payments. After five years, how much would you have in this account?

- More than $102 (correct answer),
- Exactly $102,
- Less than $102,
- Do not know,
- Refuse to answer.

b. Imagine that the interest rate on your savings account was 1% per year and inflation was 2% per year. After one year, how much would you be able to buy with the money in this account?

- More than today,
- Exactly the same,
- Less than today (correct answer),
- Do not know,
- Refuse to answer.

c. Please indicate whether the following statement is true or false. “Buying a company stock usually provides a safer return than buying a stock mutual fund”.

- True,
- False (correct answers),
- Do not know,
- Refuse to answer.

The first two questions measure the respondent’s understanding of how compound interest works and the effect of inflation. Indeed, the questions help gauge the understanding of economic concepts and basic numeracy [63]. The third question measures the respondents’ ability to understand the concept of risk diversification. In this study, we assigned a score of 1 for each correct answer and 0 for each incorrect answer. We calculated the financial literacy variable by taking the equal weight of the average scores of the three questions.

For financial education, the respondents were asked, “Did you receive any compulsory financial education when you were in high school?” with three possible responses: yes, no, and do not know. We coded the respondents who answered yes as 1 and those who answered no or do not know as 0. We treated this as a binary variable.

Furthermore, we included gender, age, university degree, marital status, household members, employment status, household income, and household assets as demographic variables in the specifications. We also controlled for risky behaviors (exercise, drinking alcohol, and gambling behavior), myopic view of the future, risk preference, level of happiness, and anxiety about health. Table 1 provides the definitions of all variables.

#### 2.1.3. Descriptive Statistics

The descriptive statistics in Table 2 show that 13.70% of the respondents were current smokers. On average, respondents’ financial literacy scores were 0.70, and 12.40% of the sample received financial education at school. For the demographic variables, about 45.29% of the sample were men and the average age was 49.30 years. Approximately 40.20% of the sample held a university degree, 63.33% were currently married, and 6.37% were divorced. The respondents had three household members on average, and about 71.97% of the sample had children. Only 2.22% of the sample was currently unemployed. Respondents had an annual household income of approximately 67,501 USD on average and 194,648 USD in household assets in 2009. For risky behaviors, 65.65% of the participants exercised regularly, while 40.38% were current drinkers and 7.62% were frequent gamblers. Overall, 10.44% of the respondents had a myopic view of the future and risk preferences of 66.35%; in other words, they were risk lovers. Respondents rated their current level of happiness at 71.10%, and 31.40% of the participants were anxious about their health.

Table 3, Table 4 and Table 5 present the distribution of smoking behavior classified by age group, demographic characteristics, and risky behaviors, respectively. Our sample contained 525 current smokers; that is, 13.70% of the total sample smoke between sometimes to more than two packs of cigarettes daily, while the remaining 3306 respondents were non-smokers. The results in Table 3 indicate significant differences in smoking behavior among age groups. The proportion of current smokers in the oldest age group (age 61 years and older) was 9.12%, which is less than that in the other age groups, and the proportion of current smokers was more than 12%. In Table 4, we see significant differences in smoking behavior between genders, education levels, and employment statuses. Approximately 14.76% of male respondents, 18.46% of respondents with education below a university degree, and 34.12% of unemployed respondents were current smokers, which is higher than their counterparts. The results in Table 5 for risky behaviors show that about 11.89% of respondents who exercise regularly are current smokers, which is less than the sample of current smokers who do not exercise regularly (17.17%). In addition, we observed considerable differences in smoking behavior between current drinkers and non-drinkers and between frequent gamblers and non-gamblers. Specifically, 18.36% of current drinkers and 23.63% of frequent gamblers were current smokers.

### 2.2. Methodology

To investigate how financial literacy and financial education are related to smoking behavior, we first estimated the effects of financial literacy and financial education separately in Equations (1) and (2), respectively. We then included both financial literacy and financial education to examine the combined effect of the variables in Equation (3).

(1) $$Y_{i}=f\left(FL_{i},X_{i},\varepsilon_{i}\right)$$

(2) $$Y_{i}=f\left(FE_{i},X_{i},\varepsilon_{i}\right)$$

(3) $$Y_{i}=f\left(FL_{i},FE_{i},X_{i},\varepsilon_{i}\right),$$

where $Y_{i}$ is the smoking behavior of the $i^{th}$ respondent (current smokers or non-smokers), *FL* represents the score on the financial literacy questions, *FE* represents financial education received at school, X is a vector of individual characteristics, and ε is the error term. Because the dependent variable is a binary choice, we employed a probit regression to estimate all equations.

As there is a potential for multicollinearity between the explanatory variables in the models (i.e., individuals with a high level of education could have high financial knowledge, or individuals with high net worth may have more financial knowledge because of their experience managing assets), we conducted correlation and multicollinearity tests in all models (available upon request). The correlation matrix shows a weak relationship between the explanatory variables (lower than 0.70). In addition, the variance inflation factor tests of the explanatory variables are below 10, indicating that multicollinearity is not significant in all models.

The full specifications of models 1, 2, and 3 are provided in the Equations (4)–(6), respectively:

(4) $$\begin{array}{cc} \mathrm{Smoking}\;{\mathrm{behavior}}_{i} & \left(1=\mathrm{current}\;\mathrm{smokers}\;\mathrm{and}\;0=\mathrm{non}\text{-}\mathrm{smokers}\right)\\ & =\beta_{0}+\beta_{1}\mathrm{financial}\;{\mathrm{literacy}}_{i}+\beta_{2}{\mathrm{male}}_{i}+\beta_{3}{\mathrm{age}}_{i}\\ & +\beta_{4}\mathrm{age}\;{\mathrm{squared}}_{i}+\beta_{5}\mathrm{university}{\mathrm{degree}}_{i}+\beta_{6}{\mathrm{marriage}}_{i}\\ & +\beta_{7}{\mathrm{divorce}}_{i}\\ & +\beta_{8}\mathrm{household}\;{\mathrm{members}}_{i}+\beta_{9}{\mathrm{children}}_{i}+\beta_{10}{\mathrm{unemployed}}_{i}\\ & +\beta_{11}\log\;\mathrm{of}\;\mathrm{household}\;{\mathrm{income}}_{i}\\ & +\beta_{12}\log\;\mathrm{of}\;\mathrm{household}\;{\mathrm{assets}}_{i}+\beta_{13}\mathrm{regular}\;{\mathrm{exercise}}_{i}\\ & +\beta_{14}\mathrm{current}\;{\mathrm{drinkers}}_{i}+\beta_{15}\mathrm{frequent}\;{\mathrm{gamblers}}_{i}\\ & +\beta_{16}\mathrm{myopic}\;\mathrm{view}\;\mathrm{of}\;\mathrm{the}\;{\mathrm{future}}_{i}\\ & +\beta_{17}\mathrm{level}\;\mathrm{of}\;\mathrm{risk}\;{\mathrm{preference}}_{i}\\ & +\beta_{18}\mathrm{current}\;\mathrm{level}\;\mathrm{of}\;{\mathrm{happiness}}_{i}\\ & +\beta_{19}\mathrm{anxiety}\;\mathrm{about}\;{\mathrm{health}}_{i}+\varepsilon_{i} \end{array}$$

(5) $$\begin{array}{cc} \mathrm{Smoking}\;{\mathrm{behavior}}_{i} & \left(1=\mathrm{current}\;\mathrm{smokers}\;\mathrm{and}\;0=\mathrm{non}\text{-}\mathrm{smokers}\right)\\ & =\beta_{0}+\beta_{1}\mathrm{financial}\;{\mathrm{education}}_{i}+\beta_{2}{\mathrm{male}}_{i}+\beta_{3}{\mathrm{age}}_{i}\\ & +\beta_{4}\mathrm{age}\;{\mathrm{squared}}_{i}+\beta_{5}\mathrm{university}{\mathrm{degree}}_{i}+\beta_{6}{\mathrm{marriage}}_{i}\\ & +\beta_{7}{\mathrm{divorce}}_{i}\\ & +\beta_{8}\mathrm{household}\;{\mathrm{members}}_{i}+\beta_{9}{\mathrm{children}}_{i}+\beta_{10}{\mathrm{unemployed}}_{i}\\ & +\beta_{11}\log\;\mathrm{of}\;\mathrm{household}\;{\mathrm{income}}_{i}\\ & +\beta_{12}\log\;\mathrm{of}\;\mathrm{household}\;{\mathrm{assets}}_{i}+\beta_{13}\mathrm{regular}\;{\mathrm{exercise}}_{i}\\ & +\beta_{14}\mathrm{current}\;{\mathrm{drinkers}}_{i}+\beta_{15}\mathrm{frequent}\;{\mathrm{gamblers}}_{i}\\ & +\beta_{16}\mathrm{myopic}\;\mathrm{view}\;\mathrm{of}\;\mathrm{the}\;{\mathrm{future}}_{i}\\ & +\beta_{17}\mathrm{level}\;\mathrm{of}\;\mathrm{risk}\;{\mathrm{preference}}_{i}\\ & +\beta_{18}\mathrm{current}\;\mathrm{level}\;\mathrm{of}\;{\mathrm{happiness}}_{i}\\ & +\beta_{19}\mathrm{anxiety}\;\mathrm{about}\;{\mathrm{health}}_{i}+\varepsilon_{i} \end{array}$$

(6) $$\begin{array}{cc} \mathrm{Smoking}\;{\mathrm{behavior}}_{i} & \left(1=\mathrm{current}\;\mathrm{smokers}\;\mathrm{and}\;0=\mathrm{non}\text{-}\mathrm{smokers}\right)\\ & =\beta_{0}+\beta_{1}\mathrm{financial}\;{\mathrm{literacy}}_{i}+\beta_{2}\mathrm{financial}\;{\mathrm{education}}_{i}\\ & +\beta_{3}{\mathrm{male}}_{i}+\beta_{4}{\mathrm{age}}_{i}+\beta_{5}\mathrm{age}\;{\mathrm{squared}}_{i}\\ & +\beta_{6}\mathrm{university}{\mathrm{degree}}_{i}+\beta_{7}{\mathrm{marriage}}_{i}+\beta_{8}{\mathrm{divorce}}_{i}\\ & +\beta_{9}\mathrm{household}\;{\mathrm{members}}_{i}+\beta_{10}{\mathrm{children}}_{i}+\beta_{11}{\mathrm{unemployed}}_{i}\\ & +\beta_{12}\log\;\mathrm{of}\;\mathrm{household}\;{\mathrm{income}}_{i}\\ & +\beta_{13}\log\;\mathrm{of}\;\mathrm{household}\;{\mathrm{assets}}_{i}+\beta_{14}\mathrm{regular}\;{\mathrm{exercise}}_{i}\\ & +\beta_{15}\mathrm{current}\;{\mathrm{drinkers}}_{i}+\beta_{16}\mathrm{frequent}\;{\mathrm{gamblers}}_{i}\\ & +\beta_{17}\mathrm{myopic}\;\mathrm{view}\;\mathrm{of}\;\mathrm{the}\;{\mathrm{future}}_{i}\\ & +\beta_{18}\mathrm{level}\;\mathrm{of}\;\mathrm{risk}\;{\mathrm{preference}}_{i}\\ & +\beta_{19}\mathrm{current}\;\mathrm{level}\;\mathrm{of}\;{\mathrm{happiness}}_{i}\\ & +\beta_{20}\mathrm{anxiety}\;\mathrm{about}\;{\mathrm{health}}_{i}+\varepsilon_{i} \end{array}$$

## 3. Results

We present the results of the probit regressions to estimate Equations (1)–(3) in Table 6, Table 7 and Table 8, respectively. Each table presents the results of the four different explanatory variable specifications. The first specification (Models 1.1, 2.1, and 3.1) included controls for only demographic variables. In the second specification (Models 1.2, 2.2, and 3.2), we added risky behaviors, including exercise, alcohol consumption, and gambling. The third specification (Models 1.3, 2.3, and 3.3) includes respondents’ myopic views of the future and risk preferences. Finally, the fourth specification (Models 1.4, 2.4, and 3.4) includes respondents’ self-rated levels of happiness and anxiety about health.

The results in Table 6 show that financial literacy has a negative and strongly significant impact on smoking behavior across the models at the 5% and 1% levels. In Table 7, financial education also has a negative impact on smoking behavior, but the coefficients are insignificant. We included both financial literacy and financial education as explanatory variables in our final model. The results in Table 8 show that, overall, there are no differences in the significance of the estimated parameters compared to the results in Table 6 and Table 7. The coefficients of financial literacy are still negative and strongly significant, while financial education is insignificant. The implication is that respondents with a high level of financial literacy were less likely to be current smokers, but we found no relationship in the case of financial education. To have an additional insight into the influence of financial literacy on smoking behavior among different groups of people, we re-estimated the models in Table 6 and Table 8 using an interaction term among financial literacy, gender, and education. The results show that the interaction variable has a significantly negative association with smoking behavior, meaning that financially literate people are less likely to be smokers when they are males and highly educated. The signs and significance of other variables remain the same. However, we did not report the re-estimation results to save on space, yet they are available upon request.

The signs and significance levels of most of the control variables are consistent across the models and specifications. Being male, having a certain age and being unemployed had a positive impact on being a current smoker. In contrast, age squared, university degree, marriage, household members, log of household income, and the log of household assets have a negative and significant relationship with the likelihood of being a current smoker (except the log of household income in Model 3.1). However, divorce and children have insignificant impacts. Regarding risky health behaviors, regular exercise has a significantly negative impact at the 5% level, while being a current drinker and frequent gambler have a positive impact on current smoking status at the 1% level of significance. Furthermore, a myopic view of the future is associated with being a current smoker but weakly significant at the 10% level in Model 3.3. Conversely, individuals with higher subjective feelings of happiness is less likely to be current smokers, and the impact is statistically significant at the 5% level. However, respondents’ risk preferences and anxiety regarding health had an insignificant impact on smoking behavior.

Endogeneity is a potential problem in our regression models, which could emerge from causality or omitted variables. To check whether our results are affected by the endogeneity problem, we used the Generalized Structural Equation Model (GSEM) in probit regression models to re-estimate the models in Table 8. The GSEM in probit model controls endogeneity by including common, unobserved components into the equations for many variables. The results of the GSEM in probit regression models show that the signs and significance of all the variables are similar to those of probit regression models, which indicate that our original results are not biased due to the endogeneity problem. However, we did not report the results of the GSEM in probit regression models to save on space, but these are available upon request.

## 4. Discussion

This study aimed to determine the relationship between financial literacy, financial education, and smoking status in the United States. Despite having negative health, behavioral, and economic consequences, why people smoke and what can potentially keep them away from smoking remain important questions. We hypothesized that financial literacy and financial education, which enable people to make rational decisions, reduce the likelihood of being smokers. In this study, we used financial literacy as a proxy for the ability to make rational decisions and used smoking behavior as a proxy for irrational decisions.

One of our major findings is that financial literacy is negatively related to smoking behavior and is statistically significant. This result is consistent with the proposition of the Grossman human capital model [33,34], in which smoking is a disinvestment in health capital and a catalyst for productivity loss and utility loss. Our result for the relationship between financial literacy and smoking behavior is similar to that reported by Watanapongvanich, Khan, Putthinun, Ono, and Kadoya [1]. Watanapongvanich, Khan, Putthinun, Ono, and Kadoya [1] found that financial literacy has a significantly negative relationship with smoking behavior in Japan. Thus, our findings confirm that financial literacy enables people to make rational decisions, and we therefore observe that financially literate people in the United States are less likely to be smokers. Moreover, the role of financial literacy in reducing the tendency to smoke prevails, irrespective of cultural differences and other country perspectives. Previous studies provided evidence that financial literacy plays a significant role in financial decision-making, regardless of cultural and country differences [73,74,75]. Our findings appear to generalize the role of financial literacy as a rational decision-making instrument to reduce irrational health risk behaviors such as smoking.

As hypothesized, our estimation results indicate no significant relationship between financial education and smoking behavior in the United States. As Watanapongvanich, Khan, Putthinun, Ono, and Kadoya [1] found that financial education is a factor contributing to the reduction in smoking behavior in Japan, the insignificant association we found for the United States requires an explanation. We argue that we do not find the hypothesized relationship between financial education and smoking behavior for three reasons. First, differences in the financial education programs and their implementation could be responsible for the insignificant association between financial education and smoking behavior in the United States. Japan implemented financial education in elementary schools, in collaboration with teachers, experts, and the government to help young students develop savings behavior and to make financial plans for their future by introducing a child bank. In contrast, the United States initiates financial education among high school students by introducing financial education in the academic curriculum, focusing mainly on how they should manage their own finances [1,76,77]. Moreover, decisions about providing financial education in high schools vary at the state and district levels and are implemented by offering optional finance-related courses. Previous studies argued that financial education programs and their implementation across countries produce different results [78]. Moreover, financial education programs did not result in the expected outcomes in New Zealand, Australia, Canada, the United Kingdom, and the United States, despite much optimism [78]. Second, cultural differences between Japan and the United States provide additional insights into the insignificant association between financial education and smoking behavior in the United States. Culture is an important element in understanding how people learn and prioritize knowledge [79]. Educational programs may not achieve their intended objectives if they do not account for cultural aspects. Giorgetti, Campbell, and Arslan [80] correctly attributed the relationship between culture and education as very close, complex, and mutually interdependent. Radhika [81] found that cultural elements such as individualism and collectivism have profound implications for educational attainment. Thus, cultural elements in the United States and their internal diversity might lessen the impact of financial education programs on changing the development of rationality in people regarding health risk behavior. Third, the measurement of financial education could be another reason why we did not find a significant association between financial education and smoking behavior. Financial education is a comprehensive issue that deals with the management of money for sound financial decisions. Thus, participation in the financial education program in the high school level, which we used as a proxy for financial education, might not appropriately measure the financial education received by the respondents.

Among the demographic characteristics, we found that males were more likely to smoke than females. This result is similar to the findings of Chinwong, Mookmanee, Chongpornchai, and Chinwong [82]; Bauer, Göhlmann, and Sinning [83]; Jha, Ranson, Nguyen, and Yach [84]; Mandil, BinSaeed, Ahmad, Al-Dabbagha, Alsaadi, and Khan [85]; and Watanapongvanich, Khan, Putthinun, Ono, and Kadoya [1], who found that smoking is more prevalent and progressive in men. The Centers of Disease Control and Prevention (CDC) [86] also found that among adults in 2019, about 15 of every 100 adult men smoke compared to 13 of every 100 adult women. Besides gender, age has a nonlinear positive relationship with smoking behavior, meaning that people tend to be frequent smokers up to a certain age, after which the tendency begins to lessen. This finding is supported by those of Çiftci, Ayoz, Baygul, Onen, and Sen [87] and Mandil, BinSaeed, Ahmad, Al-Dabbagha, Alsaadi, and Khan [85]. According to the CDC [86], current cigarette smoking was the highest among people aged 25 to 64 years. We also found a relationship between marital status and smoking behavior, in that married respondents were less likely to be smokers. Our result is consistent with the finding that the smoking rate is higher among people who are not currently married, separated, and divorced [1,86,88,89,90].

Among the socio-economic characteristics, a higher level of education is negatively related to smoking behavior among the people in the United States, meaning that those who attained a higher level of education were less likely to be frequent smokers. Our finding is consistent with previous studies and could be explained by the proposition that highly educated people are aware of the negative health consequences of smoking because of their knowledge and access to smoking cessation services [91,92,93]. Apart from being less educated, people who are unemployed have a higher likelihood of smoking in the United States. This result is consistent with Okechukwu, Bacic, Cheng, and Catalano’s [94] study that found a positive association between smoking and unemployment in the United States using the 2006–07 U.S. Current Population Survey. However, this result contradicts the results reported by Watanapongvanich, Khan, Putthinun, Ono, and Kadoya [1], who found no significant association between unemployment status and smoking behavior in Japan. Socio-economic phenomena such as household income, household assets, and household members were negatively related to smoking behavior in the United States. While the overall prevalence of smoking has declined over the last 20 years due to an increase in anti-smoking advertisements, access to cessation interventions, and taxes on cigarettes, the evidence suggests that smoking disproportionately affects individuals of a lower socioeconomic status [1,95].

For the variables related to health risk behavior, both current drinking and frequent gambling status had a significantly positive association with smoking behavior, whereas regular exercise was negatively associated with smoking behavior. Findings from previous studies [96,97,98,99,100] also support our results. These authors argue that drinkers raise their rate of smoking and gambling encourages people to smoke more during a game. We can plausibly explain this result that health-conscious people tend to exercise regularly to maintain their health and avoid health-deteriorating behavior to avoid any adverse effects. In contrast, individuals possessing risky health behaviors such as drinking or gambling are more likely to be smokers. In summary, when people behave rationally, they tend to care more about their health and avoid irrational health-risk behavior.

Finally, respondents in the United States with self-reported higher current levels of happiness were less likely to smoke. These results are similar to those of previous studies [1,101,102] that when people are in a happier state, they are less likely to behave irrationally. Chang, Chu, Deale, and Gupta [101] found that happy people in Japan, France, and the United Kingdom smoked less. In contrast, people experiencing stress are more likely to behave irrationally, believing that smoking makes them feel more relaxed and energetic. Previous studies also found that people sometimes use smoking as a coping mechanism to combat stress, as it gives them pleasure [102,103].

## 5. Conclusions

Using the PPS of Osaka University conducted in the United States in 2010, we examined whether financial literacy and financial education influence smoking behavior in the United States. Our sample consisted of 3831 individuals from the District of Columbia and 48 other states (except Alaska and Hawaii). We hypothesized that respondents with financial literacy and financial education will be more conscious in making irrational decisions such as smoking. Using probit models, our study shows that the relationship between financial literacy and smoking behavior is significantly negative, meaning that financially literate people are less likely to be smokers. We argue that financial literacy is a rational decision-making instrument that protects people from irrational behaviors such as smoking. However, we found no significant relationship between financial education and smoking behavior, although we believe that financial education is a rational decision-making instrument. This finding contradicts Watanapongvanich, Khan, Putthinun, Ono, and Kadoya [1], who found that financial education in Japan reduced the likelihood of smoking. We argue that the nature of financial education programs and their implementation varies across countries, and they may therefore not achieve their expected outcomes. Moreover, cultural differences sometimes make the expected outcome of financial education less certain. Our results also show that respondents’ gender, age, unemployment status, and risky health behaviors such as drinking and gambling are positive predictors of being a current smoker. In contrast, marital status, university degree, family size, household income, household assets, physical exercise, and happiness level were negative predictors of current smoking status.

Given that smoking is still a predominant social problem, our findings provide a new perspective on this social problem, enabling people with rational decision-making abilities to reduce irrational behavior such as smoking. Our findings are of significant importance from a regulatory perspective. The results justify and reinforce the efforts that promote financial literacy as a rational decision-making behavior that could help mitigate irrational decision-making behavior such as smoking. Our findings also suggest that authorities should take comprehensive actions to review existing financial education programs, find loopholes, and formulate new strategies to ensure that financial education programs deliver their expected outcomes by helping people become rational decision makers. As a direction for future research, we suggest conducting a comprehensive study to examine whether financial literacy and financial education as rational decision-making instruments reduce the likelihood of engaging in other risky health behaviors.

This study has some limitations. First, we measured financial literacy using three questions, following Lusardi and Mitchell [63]. Although alternative measurements of financial literacy exist, we used this method to ensure international comparability [1]. Second, our smoking status variable considers only participation in smoking, not the volume of smoking due to limited data availability. Third, we had to exclude several respondents due to missing data on some financial, socio-economic, risky health behavior questions. We believe that the excluded sample would not have a significant impact on our results because the exclusion was not made on purpose and did not have a specific pattern. Despite these limitations, this study provides empirical evidence suggesting that rational decision-making abilities, generated by enhancing financial literacy, can be a tool to reduce irrational behavior such as smoking. Additionally, because the United States exhibits the typical smoking behavior found in developed countries, our results can be generalized to other developed countries.

## Figures and Tables

**Table 1:** Variable definitions.

Variable	Definition
Smoking behavior	Binary variable: 1 = current smoker (sometimes–more than two packs a day) and 0 = non-smokers (do not smoke at all, quit, or hardly smoke)
Financial literacy	Continuous variable: number of correct answers from three financial literacy questions
Financial education	Binary variable: 1 = received compulsory financial education at school and 0 = otherwise
Male	Binary variable: 1 = male and 0 = female
Age	Respondent’s age
Age squared	Age squared
University degree	Binary variable: 1 = obtained university degree and 0 = otherwise
Marriage	Binary variable: 1 = married and 0 = otherwise
Divorce	Binary variable: 1 = divorced or separated and 0 = otherwise
Household members	Continuous variable: number of people currently living in the household
Children	Binary variable: 1 = have child/children and 0 = otherwise
Unemployed	Binary variable: 1 = respondent is unemployed and 0 = otherwise
Household income	Continuous variable: annual earned income before taxes and with bonuses of the entire household in 2009 (unit: USD)
Log of household income	Log (household income)
Household assets	Continuous variable: balance of financial assets (savings, stocks, insurance, etc.) of the entire household (unit: USD)
Log of household assets	Log (household assets)
Regular exercise	Binary variable: 1 = regular exercise (exercise once a week or more) and 0 = otherwise
Current drinker	Binary variable: 1 = current drinker (drink sometimes—five cans of beer daily) and 0 = otherwise
Frequent gambler	Binary variable: 1 = frequent gambler (gamble once a week or more) and 0 = otherwise
Myopic view of the future	Binary variable: 1 = agree and completely agree with the statement “Since the future is uncertain, it is a waste to think about it” and 0 = otherwise
Level of risk preference	Continuous variable: percentage score from the question “Usually, when you go outdoors, how high does the probability of rain have to be before you take an umbrella?”
Current level of happiness	Continuous variable: percentage score from the question “Overall, how happy would you say you are currently?”
Anxiety about health	Binary variable: 1 = agree and completely agree with the statement “I have anxiety about my health” and 0 = otherwise

**Table 2:** Descriptive statistics.

Variable	Mean	Standard Deviation (SD)	Min	Max
Smoking behavior	0.1370	0.3439	0	1
Financial literacy	0.6981	0.3155	0	1
Financial education	0.1240	0.3296	0	1
Male	0.4529	0.4978	0	1
Age	49.30	15.89	15	96
Age squared	2683.34	1625.95	225	9216
University degree	0.4020	0.4904	0	1
Marriage	0.6333	0.4820	0	1
Divorce	0.0637	0.2442	0	1
Household members	2.79	1.47	1	13
Children	0.7197	0.4492	0	1
Unemployed	0.0222	0.1473	0	1
Household income	67,501.96	48,617.02	5000	210,000
Log of household income	10.77	0.95	8.52	12.25
Household assets	194,648.90	292,005.20	12,500	1,250,000
Log of household assets	11.14	1.49	9.43	14.04
Regular exercise	0.6565	0.4749	0	1
Current drinker	0.4038	0.4907	0	1
Frequent gambler	0.0762	0.2654	0	1
Myopic view of future	0.1044	0.3058	0	1
Level of risk preference	0.6635	0.2843	0	0.99
Current level of happiness	0.7110	0.2231	0	1
Anxiety about health	0.3140	0.4642	0	1
Observations	3831			

**Table 3:** Distribution of smoking behavior by age group.

Smoking Behavior	Age					Total
	≤30	31–40	41–50	51–60	≥61	
Non-smoker	474	496	719	780	837	3306
	84.79%	87.32%	83.51%	84.60%	90.88%	86.30%
Current smoker	85	72	142	142	84	525
	15.21%	12.68%	16.49%	15.40%	9.12%	13.70%
Total	559	568	861	922	921	3831
	100%	100%	100%	100%	100%	100%
Mean difference	F = 6.50 ***					

Note: *** *p* < 0.01.

**Table 4:** Distribution of smoking behavior by demographic characteristic.

Smoking Behavior	Gender		Education		Unemployed		Total
	Female	Male	Less Than University Degree	University Degree and Higher	No	Yes	
Non-smoker	1827	1479	1868	1438	3250	56	3306
	87.17%	85.24%	81.54%	93.38%	86.76%	65.88%	86.30%
Current smoker	269	256	423	102	496	29	525
	12.83%	14.76%	18.46%	6.62%	13.24%	34.12%	13.70%
Total	2096	1735	2291	1540	3746	85	3831
	100%	100%	100%	100%	100%	100%	100%
Mean difference	t = −1.7213 *		t = 10.5979 ***		t = −5.5554 ***		

Note: *** *p* < 0.01, * *p* < 0.10.

**Table 5:** Distribution of smoking behavior by risky behavior.

Smoking Behavior	Regular Exercise		Current Drinker		Frequent Gambler		Total
	No	Yes	No	Yes	No	Yes	
Non-smoker	1090	2216	2043	1263	3083	223	3306
	82.83%	88.11%	89.45%	81.64%	87.12%	76.37%	86.30%
Current smoker	226	299	241	284	456	69	525
	17.17%	11.89%	10.55%	18.36%	12.88%	23.63%	13.70%
Total	1316	2515	2284	1547	3539	292	3831
	100%	100%	100%	100%	100%	100%	100%
Mean difference	t = 4.5277 ***		t = −6.9354 ***		t = −5.1482 ***		

Note: *** *p* < 0.01.

**Table 6:** Probit model regression results: financial literacy as the main explanatory variable.

Variable	Dependent Variable: Smoking Behavior			
	Model 1.1	Model 1.2	Model 1.3	Model 1.4
Financial literacy	−0.226 **	−0.240 ***	−0.233 ***	−0.230 **
	(0.0887)	(0.0894)	(0.0897)	(0.0899)
Male	0.189 ***	0.124 **	0.124 **	0.122 **
	(0.0554)	(0.0568)	(0.0569)	(0.0570)
Age	0.0613 ***	0.0623 ***	0.0624 ***	0.0601 ***
	(0.0109)	(0.0113)	(0.0114)	(0.0114)
Age squared	−0.000687 ***	−0.000685 ***	−0.000688 ***	−0.000664 ***
	(0.000109)	(0.000113)	(0.000114)	(0.000114)
University degree	−0.500 ***	−0.504 ***	−0.501 ***	−0.500 ***
	(0.0635)	(0.0641)	(0.0643)	(0.0645)
Marriage	−0.190 **	−0.183 **	−0.181 **	−0.172 **
	(0.0790)	(0.0797)	(0.0798)	(0.0801)
Divorce	0.0714	0.0539	0.0555	0.0326
	(0.117)	(0.118)	(0.118)	(0.119)
Household members	−0.0523 **	−0.0419 *	−0.0418 *	−0.0426 *
	(0.0241)	(0.0244)	(0.0245)	(0.0247)
Children	0.111	0.0956	0.0933	0.108
	(0.0831)	(0.0837)	(0.0839)	(0.0843)
Unemployed	0.484 ***	0.497 ***	0.497 ***	0.471 ***
	(0.152)	(0.151)	(0.152)	(0.151)
Log of household income	−0.0462	−0.0694 **	−0.0653 *	−0.0602 *
	(0.0338)	(0.0343)	(0.0343)	(0.0345)
Log of household assets	−0.0779 ***	−0.0842 ***	−0.0841 ***	−0.0806 ***
	(0.0223)	(0.0227)	(0.0227)	(0.0228)
Regular exercise		−0.128 **	−0.128 **	−0.110 **
		(0.0556)	(0.0557)	(0.0560)
Current drinker		0.437 ***	0.440 ***	0.445 ***
		(0.0560)	(0.0560)	(0.0560)
Frequent gambler		0.382 ***	0.378 ***	0.371 ***
		(0.0905)	(0.0905)	(0.0906)
Myopic view of the future			0.147 *	0.132
			(0.0815)	(0.0817)
Level of risk preference			−0.115	−0.113
			(0.0911)	(0.0911)
Current level of happiness				−0.294 **
				(0.120)
Anxiety about health				0.00389
				(0.0591)
Constant	−0.564	−0.432	−0.425	−0.288
	(0.393)	(0.398)	(0.402)	(0.408)
Observations	3831	3831	3831	3831
Log likelihood	−1404	−1361	−1358	−1355
Chi^2^ statistics	222.1	289.4	293.2	301.4
*p*-value	0	0	0	0

Note: Robust standard errors are in parentheses; *** *p* < 0.01, ** *p* < 0.05, * *p* < 0.1.

**Table 7:** Probit model regression results: financial education as the main explanatory variable.

Variable	Dependent Variable: Smoking Behavior			
	Model 2.1	Model 2.2	Model 2.3	Model 2.4
Financial education	−0.00865	−0.0111	−0.0135	−0.00644
	(0.0828)	(0.0843)	(0.0845)	(0.0848)
Male	0.176 ***	0.110 *	0.111 **	0.110 *
	(0.0550)	(0.0564)	(0.0566)	(0.0567)
Age	0.0598 ***	0.0605 ***	0.0607 ***	0.0583 ***
	(0.0109)	(0.0113)	(0.0113)	(0.0114)
Age squared	−0.000673 ***	−0.000669 ***	−0.000673 ***	−0.000648 ***
	(0.000109)	(0.000113)	(0.000114)	(0.000114)
University degree	−0.527 ***	−0.532 ***	−0.528 ***	−0.526 ***
	(0.0620)	(0.0627)	(0.0629)	(0.0631)
Marriage	−0.190 **	−0.184 **	−0.181 **	−0.172 **
	(0.0789)	(0.0796)	(0.0797)	(0.0800)
Divorce	0.0678	0.0508	0.0524	0.0298
	(0.117)	(0.118)	(0.118)	(0.119)
Household members	−0.0511 **	−0.0406 *	−0.0404 *	−0.0413 *
	(0.0237)	(0.0241)	(0.0242)	(0.0244)
Children	0.119	0.103	0.0999	0.115
	(0.0826)	(0.0832)	(0.0834)	(0.0838)
Unemployed	0.487 ***	0.500 ***	0.499 ***	0.473 ***
	(0.152)	(0.152)	(0.152)	(0.152)
Log of household income	−0.0538	−0.0768 **	−0.0723 **	−0.0671 *
	(0.0335)	(0.0340)	(0.0341)	(0.0343)
Log of household assets	−0.0888 ***	−0.0956 ***	−0.0952 ***	−0.0914 ***
	(0.0219)	(0.0223)	(0.0222)	(0.0224)
Regular exercise		−0.132 **	−0.131 **	−0.114 **
		(0.0559)	(0.0560)	(0.0563)
Current drinker		0.431 ***	0.434 ***	0.439 ***
		(0.0559)	(0.0560)	(0.0559)
Frequent gambler		0.386 ***	0.382 ***	0.374 ***
		(0.0907)	(0.0907)	(0.0909)
Myopic view of the future			0.153 *	0.138 *
			(0.0812)	(0.0814)
Level of risk preference			−0.120	−0.119
			(0.0910)	(0.0910)
Current level of happiness				−0.297 **
				(0.120)
Anxiety about health				0.00633
				(0.0591)
Constant	−0.466	−0.331	−0.325	−0.190
	(0.390)	(0.395)	(0.399)	(0.405)
Observations	3831	3831	3831	3831
Log likelihood	−1407	−1364	−1362	−1359
Chi^2^ statistics	214.7	282.7	285.8	293.8
*p*-value	0	0	0	0

Note: Robust standard errors are in parentheses; *** *p* < 0.01, ** *p* < 0.05, * *p* < 0.1.

**Table 8:** Probit model regression results: financial literacy and financial education as the main explanatory variables.

Variable	Dependent Variable: Smoking Behavior			
	Model 3.1	Model 3.2	Model 3.3	Model 3.4
Financial literacy	−0.226 **	−0.241 ***	−0.233 ***	−0.231 **
	(0.0888)	(0.0896)	(0.0900)	(0.0902)
Financial education	0.00708	0.00582	0.00280	0.00952
	(0.0829)	(0.0844)	(0.0846)	(0.0849)
Male	0.189 ***	0.123 **	0.124 **	0.122 **
	(0.0554)	(0.0568)	(0.0569)	(0.0570)
Age	0.0614 ***	0.0623 ***	0.0625 ***	0.0601 ***
	(0.0109)	(0.0113)	(0.0113)	(0.0114)
Age squared	−0.000687 ***	−0.000685 ***	−0.000689 ***	−0.000664 ***
	(0.000109)	(0.000113)	(0.000114)	(0.000114)
University degree	−0.500 ***	−0.503 ***	−0.501 ***	−0.499 ***
	(0.0635)	(0.0641)	(0.0643)	(0.0645)
Marriage	−0.190 **	−0.183 **	−0.181 **	−0.172 **
	(0.0792)	(0.0799)	(0.0800)	(0.0803)
Divorce	0.0716	0.0541	0.0556	0.0328
	(0.117)	(0.118)	(0.118)	(0.119)
Household members	−0.0524 **	−0.0419 *	−0.0418 *	−0.0426 *
	(0.0241)	(0.0244)	(0.0245)	(0.0247)
Children	0.111	0.0957	0.0934	0.108
	(0.0830)	(0.0837)	(0.0839)	(0.0842)
Unemployed	0.485 ***	0.497 ***	0.497 ***	0.471 ***
	(0.152)	(0.152)	(0.152)	(0.152)
Log of household income	−0.0463	−0.0695 **	−0.0654 *	−0.0603 *
	(0.0338)	(0.0343)	(0.0344)	(0.0345)
Log of household assets	−0.0779 ***	−0.0842 ***	−0.0841 ***	−0.0805 ***
	(0.0223)	(0.0227)	(0.0227)	(0.0228)
Regular exercise		−0.129 **	−0.128 **	−0.111 **
		(0.0559)	(0.0561)	(0.0564)
Current drinker		0.437 ***	0.439 ***	0.445 ***
		(0.0560)	(0.0560)	(0.0560)
Frequent gambler		0.382 ***	0.378 ***	0.371 ***
		(0.0905)	(0.0905)	(0.0906)
Myopic view of the future			0.147 *	0.132
			(0.0815)	(0.0817)
Level of risk preference			−0.115	−0.113
			(0.0911)	(0.0911)
Current level of happiness				−0.294 **
				(0.120)
Anxiety about health				0.00393
				(0.0591)
Constant	−0.564	−0.432	−0.425	−0.288
	(0.393)	(0.398)	(0.402)	(0.408)
Observations	3831	3831	3831	3831
Log likelihood	−1404	−1361	−1358	−1355
Chi^2^ statistics	222.6	289.7	293.4	301.7
*p*-value	0	0	0	0

Note: Robust standard errors are in parentheses; *** *p* < 0.01, ** *p* < 0.05, * *p* < 0.1.

## Data Availability

Data is available on request.
